# Epidemiology of Candidemia in Kuwait: A Nationwide, Population-Based Study

**DOI:** 10.3390/jof7080673

**Published:** 2021-08-20

**Authors:** Khaled Alobaid, Suhail Ahmad, Mohammad Asadzadeh, Eiman Mokaddas, Noura Al-Sweih, Khalifa Albenwan, Wadha Alfouzan, Inaam Al-Obaid, Ahlam Jeragh, Ebtihal Al-Roomi, Ziauddin Khan, Leena Joseph, Soumya Varghese

**Affiliations:** 1 Microbiology Department, Mubarak Al-Kabeer Hospital, Jabriya 46300, Kuwait; khaled22m@live.com (K.A.); Soumya.mary84@gmail.com (S.V.); 2 Department of Microbiology, Faculty of Medicine, Kuwait University, Safat 13110, Kuwait; mohammad.assadzadeh@ku.edu.kw (M.A.); eiman.mokaddas@ku.edu.kw (E.M.); nourah.alsuwaih@ku.edu.kw (N.A.-S.); khalifa.albenwan@ku.edu.kw (K.A.); alfouzan.w@ku.edu.kw (W.A.); ziauddin381044@gmail.com (Z.K.); leena.anniejoseph@ku.edu.kw (L.J.); 3 Microbiology Department, Ibn-Sina Hospital, Shuwaikh 70031, Kuwait; 4 Microbiology Department, Maternity Hospital, Shuwaikh 70031, Kuwait; 5 Microbiology Department, Amiri Hospital, Sharq 15300, Kuwait; 6 Microbiology Department, Farwaniya Hospital, Farwaniya 13373, Kuwait; 7 Microbiology Department, Al-Sabah Hospital, Shuwaikh 70031, Kuwait; inaob218@hotmail.com; 8 Microbiology Department, Adan Hospital, Hadiya 47000, Kuwait; micro_ahlam@hotmail.com; 9 Microbiology Department, Jahra Hospital, Jahra 01753, Kuwait; DrEalroomi@gmail.com

**Keywords:** candidemia, Kuwait, incidence, species spectrum, antifungal susceptibility, rare *Candida*/yeast species, treatment, outcome

## Abstract

The *Candida* species cause a majority of invasive fungal infections. In this article, we describe the nationwide epidemiology of candidemia in Kuwait in 2018. Yeast bloodstream isolates submitted from all major hospitals and identified by phenotypic MALDI-TOF MS and/or by molecular methods were studied. Susceptibility testing was performed by Etest. Out of 313 bloodstream yeasts, 239 *Candida* spp. isolates (excluding duplicate isolates) were obtained during 234 candidemic episodes among 223 patients. Mixed-species candidemia and re-infection occurred in 5 and 11 patients, respectively. *C. albicans* (*n* = 74), *C. parapsilosis* (*n* = 54), *C. tropicalis* (*n* = 35), *C. auris* (*n* = 33), *C. glabrata* (*n* = 32), other *Candida* spp. (*n* = 11), and other yeasts (*n* = 9) caused fungemia. Nearly 50% of patients were in intensive care units. *Candida* spp. isolates (except *C. glabrata*) were susceptible to caspofungin and 27% of *C. auris* were amphotericin B-resistant. Resistance to fluconazole was 100% in *C. auris*, 17% in *C. parapsilosis,* 12% in *C. glabrata,* and 1% in *C. albicans*. Mortality was 47% for other *Candida*/yeast infections. Nationwide candidemia incidence in 2018 was 5.29 cases/100,000 inhabitants. Changes in species spectrum, increasing fluconazole resistance in *C. parapsilosis,* and the emergence of *C. auris* as a major pathogen in Kuwait are noteworthy findings. The data could be of help in informing decisions regarding planning, in the allocation of resources, and in antimicrobial stewardship.

## 1. Introduction

Invasive fungal infections (IFIs) have increased significantly in the last few decades, coinciding with a concomitant increase in the population of immunocompromised/immunosuppressed individuals [1,2]. The spectrum of fungi causing IFIs is changing due to changes in clinical practice [3]. *Candida* infections constitute a major component of healthcare-associated IFIs and are associated with 20–40% of all-cause mortality, with an attributable mortality of 15–35% in adults and 10–15% in neonates [4,5,6,7,8,9]. Nearly 50% of episodes of candidemia occur in intensive care units (ICUs) and contribute towards prolonged hospital stay and considerable health expenditures [10,11].

Previous epidemiological studies have shown that nearly 90% of all *Candida* infections are caused by only four species/species complexes, which include *Candida* *albicans,* *Candida* *tropicalis,* *Candida* *parapsilosis,* and *Candida* *glabrata* [8,12,13,14]. Among *Candida* species, *C. albicans* is considered the most pathogenic and the most frequent cause of candidemia worldwide [15,16]. However, the past two decades have seen a gradual change in the spectrum of species causing candidemia, so much so that >50% of candidemia cases are now caused by non-*albicans Candida* species, which mainly include *C. glabrata* complex members, *C. parapsilosis* complex members, and *C. tropicalis* as well as many emerging pathogens, likely as a result of the increasing use of fluconazole or other antifungal drugs for prophylaxis or therapy [6,14,17,18]. *Candida* non-*albicans* species are associated with even higher rates of mortality and a higher frequency of resistance to antifungal drugs than what is seen with *C. albicans* infections [6,7,14,19,20,21]. More recently, the emergence and rapid spreading of the often multidrug-resistant *Candida auris* has dramatically changed the epidemiology of candidemia at many geographical locations/health care facilities as it has become the most common or one of the more common causes of invasive *Candida* infections [22].

The annual incidence of candidemia is quite variable in different populations and age groups, with *C. albicans* causing most infections in younger (<18 years old) patients and *C. glabrata* infections occurring more frequently among the elderly (>60 years old) [8,17,23,24,25]. The distribution of candidemia cases caused by major *Candida* spp. also varies in population-based studies carried out in different countries/geographical settings [6,8,17,23]. Although *C. albicans* is the most frequently isolated species, the number of candidemia cases caused by *C. parapsilosis, C. glabrata,* and *C. tropicalis* vary considerably, while *C. auris* has recently become a major pathogen in some healthcare facilities/geographical locations [5,6,7,8,17,22,23]. *C. glabrata* is the second, while *C. parapsilosis* is the third most frequently isolated species from candidemia patients in North America, Australia, and some European countries [5,6,7,8,17,23]. The isolation frequency of *C. glabrata* is particularly higher from patients who are critically ill, have diabetes or exposure to azoles, or have a solid organ transplant or a solid tumor [5,6,8,17,24]. *C. parapsilosis* is the second most frequently isolated species from candidemia patients in Spain, Latin America, and Africa, and its isolation frequency is higher from patients with indwelling catheters, parenteral nutrition, or prior exposure to antifungal drugs or corticosteroids [6,8,14,17,24]. *C. parapsilosis* invasive infections are more common in younger individuals, and nearly 34% of all neonatal *Candida* infections worldwide are caused by *C. parapsilosis* [6,8,26]. *C. tropicalis* invasive infections are more common in some Asian settings/countries and patients with these infections are more likely to have hematologic malignancy, neutropenia, or exposure to corticosteroids [6,8,17,24]. *C. auris* has caused invasive infections and outbreaks in more than 45 countries worldwide and unlike other *Candida* spp., which predominantly originate from the gastrointestinal tract, it readily colonizes the skin, is shed into the environment, and is easily transmitted to other hospitalized patients [8,22]. *C. auris* mostly causes invasive infections in older, critically ill patients with indwelling catheters, multiple comorbidities, and prolonged hospitalization [27,28]. Although many epidemiologic studies on invasive *Candida* infections have been performed, only a few nationwide studies have determined the incidence of candidemia [6,14,29]. This retrospective study determined the countrywide incidence of candidemia in Kuwait in 2018. The study also identified different *Candida* species that cause bloodstream infections and their resistance to commonly used antifungal drugs.

## 2. Materials and Methods

### 2.1. Candidemia Surveillance and Incidence of Candidemia in Kuwait

This retrospective, laboratory-based study performed a nationwide surveillance of candidemia cases in Kuwait from 1 January to 31 December 2018. The study collected data from 8 major and 4 tertiary care hospitals (representing all government hospitals where candidemia patients are treated in Kuwait) that routinely submit *Candida* and other yeast bloodstream isolates to the Mycology Reference Laboratory (MRL) for species-specific identification and antifungal drug susceptibility testing. Until the year 2018, all governmental hospitals were distributed over six major medical areas around the country, namely: Capital (central, sea side), Hawally (central, east side), Jahra (north), Ahmadi (south), Farwaniya (central), and the specialized Al-Sabah (central, north side) medical areas. Each major hospital is located in a different medical area, while the tertiary care hospitals are located in the specialized Al-Sabah medical area. The number of hospital beds, catchment area, and various specialties available in these hospitals are shown in Table 1.

Patients’ demographic data and the location of care at the onset of candidemia (or fungemia for other yeasts) were also recorded. The blood samples were obtained from each patient after obtaining verbal consent only as part of routine patient care and diagnostic work-up. For each patient, the first blood culture isolate growing the *Candida* species during the study period indicated an episode of candidemia. Positive blood cultures yielding the same *Candida* species within 30 days of the first isolation were attributed to the same candidemia episode and were not included. Recurrent candidemia (re-infection) was defined as a positive blood culture occurring at least one month after the previous episode of candidemia. Mixed candidemia cases indicated the presence of more than one *Candida* species during the same episode. The data on the total population of Kuwait in 2018 were obtained from the Public Authority for Civil Information (https://www.csb.gov.kw/Pages/Statistics_en?ID=67&ParentCatID=1, accessed on 14 July 2021) and was used to determine the candidemia incidence (candidemia cases/100,000 population) in Kuwait.

### 2.2. Isolation and Species-Specific Identification of Yeast Isolates

The microbiology laboratories of all government hospitals use automated blood culture systems, including BACTEC 9240 (Becton Dickinson, Sparks, MD, USA), BacT/Alert 3D (bioMérieux, Marcy-l’E’toile, France), and/or Versa TREK^TM^240 (Thermo Fisher Scientific, Waltham, MA, USA) for the isolation of yeasts from blood specimens. All growth-positive blood cultures received in the MRL were subcultured on Sabouraud dextrose agar and Mast ID-CHROMagar Candida (Mast Diagnostics, Merseyside, UK) for phenotypic colony characteristics, as described previously [30]. Species-specific identification was achieved through assimilation profiles obtained by commercial VITEK 2 yeast identification system and/or through protein profiles by MALDI-TOF MS (VITEK^®^ MS) (bioMérieux, Marcy-l’Étoile, France), as described previously [31,32]. A multiplex PCR assay was used to differentiate *C. parapsilosis* sensu stricto from *C. orthopsilosis* and *C. metapsilosis*, as described previously [33]. PCR amplification of rDNA was used to confirm the identification of all *C. auris* and *C. lusitaniae* isolates by using species-specific primers, as described previously [30,34]. The identity of selected isolates and other isolates which showed unusual phenotypic characteristics and/or resistance to antifungal drugs was confirmed by PCR sequencing of the internal transcribed spacer (ITS) region of rDNA by using panfungal primers, as described previously [35].

### 2.3. Antifungal Drug Susceptibility Testing (AST) and the Molecular Basis of Drug Resistance

The in vitro susceptibility to four (fluconazole, voriconazole, amphotericin B, and caspofungin) antifungal drugs was determined by using Etest strips (bioMérieux, Marcy l’Etoile, France) and the data were interpreted according to the manufacturer’s instructions. *C. krusei* ATCC 6258 and *C. parapsilosis* ATCC 22019 were used as reference strains for the purpose of quality control. Minimum inhibitory concentration (MIC) values were interpreted as susceptible, intermediate/susceptible dose-dependent, or resistant according to Clinical and Laboratory Standard Institute (CLSI) susceptibility breakpoints (supplement M60) [36]. If an isolate scored as resistant or intermediate to caspofungin, it was also tested against micafungin to confirm the results. Due to the lack of defined breakpoints, isolates showing an MIC ≤ 1.0 μg/mL for amphotericin B were taken as wild-type, and isolates with MIC > 1 μg/mL were scored as non-wild-type [37]. Since there are no established *C. auris*-specific susceptibility breakpoints, tentative MIC breakpoints of ≥32 µg/mL for fluconazole, ≥2 µg/mL for voriconazole, ≥4 µg/mL for caspofungin, and ≥2 µg/mL for amphotericin B (Etest MIC of 1.5 µg/mL was rounded off to 2.0 µg/mL), based on expert opinion, were used [22,38].

The molecular basis of resistance to fluconazole in *C. albicans* isolates was determined by sequence analysis of the *ERG11* gene. The complete *ERG11* gene (1578 bp) and the flanking 5′ and 3′ regions were amplified as 5 overlapping fragments and sequenced. The N-terminal fragment, internal fragments 1, 2, and 3, and the C-terminal fragment were amplified and sequenced by using the CalERG11F1 + CalERG11R1, CalERG11F2 + CalERG11R2, CalERG11F3 + CalERG11R3, CalERG11F4 + CalERG11R4, and CalERG11F5 + CalERG11R5 primers (Table 2), respectively, with the reaction and cycling conditions, as described previously [39].

The complete *ERG11* sequence was assembled and compared with the reference sequence from the fluconazole-susceptible *C. albicans* strain SC5314 (GenBank accession no. X13296). The presence of the Y132F mutation in *ERG11* in *C. parapsilosis* or the presence of the Y132F or K143R mutation in *ERG11* in *C. auris* isolates was determined, as described previously [39,40]. The *C. glabrata* isolate, which showed reduced susceptibility to caspofungin by Etest was analyzed for hotspot-1 (HS-1) mutations in the *FKS1* and *FKS2* genes [41]. The HS-1 regions of the *FKS1* and *FKS2* genes were amplified and sequenced, as described previously [41].

### 2.4. Statistical Analyses

The incidence of candidemia was calculated as the number of candidemia cases per 1,000,000 population. Patients’ demographic data and other variables were compared by the Pearson’s Chi-square test or Fisher’s exact test, as appropriate. Statistical analyses were carried out by using WinPepi software ver. 11.65 (PEPI for Windows, Microsoft Inc., Redmond, WA, USA). A *p* value < 0.05 with the use of a two-tailed test was considered statistically significant.

## 3. Results

### 3.1. Epidemiology of Candidemia in Kuwait in 2018

A total of 313 bloodstream yeast isolates were submitted to MRL from January to December 2018. Excluding duplicate isolates and non-*Candida* yeasts, 239 *Candida* spp. isolates were obtained during 234 candidemic episodes among 223 patients. The demographic details and the hospital unit housing the candidemia patients in 8 major and 4 tertiary care hospitals are shown in Table 3. The number of male patients (*n* = 122) was slightly higher than females (*n* = 100) (Table 1). Age distribution was bimodal, mainly involving extremes of age as neonates/infants (<1-year old, *n* = 51) and senior citizens (≥65 years old, *n* = 87) were the dominant age groups. The nationality data were available for only 65 patients and included 55 Kuwaiti nationals and 10 non-Kuwaiti patients (2 patients from Saudi Arabia and 1 patient each from United Arab Emirates, Syria, Jordan, Egypt, India, Nepal, Bangladesh, and Sri Lanka). Nearly half (*n* = 119) of the patients were in the ICU, but their proportion was significantly different among the hospitals (Table 1). All neonates/infants in the Maternity Hospital were in the neonatal ICUs. Excluding the Adan, Jahra, and Farwaniya hospitals, which also have maternity wards and neonatal ICUs, the number of patients located in the ICU was lower in Amiri Hospital or significantly lower (*p* < 0.05) in Mubarak Al-Kabeer Hospital compared to the number of candidemia patients in Al-Sabah Hospital or Ibn-Sina Hospital (Table 3). The incidence of candidemia in Kuwait in 2018 was determined as 5.29 cases per 100,000 inhabitants (234 candidemia episodes/4.42 million inhabitants). The incidence was slightly higher in females (6.18/100,000) than in males (4.71/100,000). The highest incidence was detected among infants/neonates (89.1/100,000), followed by the elderly (≥65-year-old group) (62.46/100,000), the 50–64-year-old group (3.81/100,000), and the >1–19-year-old group (1.99/100,000); the lowest incidence (1.69/100,000) was found in the 20–49-year-old group. Nearly 10% of the affected neonates were twins.

The spectrum of *Candida* spp. isolated during 234 candidemia episodes are shown in Table 4. Candidemia due to two species (*C. albicans + C. tropicalis*, *n* = 3; *C. albicans + C. parapsilosis, n* = 1; and *C. glabrata + C. krusei*, *n* = 1) occurred simultaneously in five patients. Eleven patients experienced re-infection at least 30 days after the onset of the first candidemic event; eight patients had re-infection with the same species (*C. auris*, *n* = 3; *C. parapsilosis*, *n* = 2; *C. tropicalis*, *n* = 2; and *C. glabrata*, *n* = 1), while three patients had re-infection with a different species (*C. krusei* followed by *C. glabrata*, *n* = 1; *C. albicans* followed by *C. auris*, *n* = 1; and *C. glabrata* followed by *C. auris*, *n* = 1). Among 239 *Candida* bloodstream isolates, *C. albicans* was detected in 74 out of 239 (31%) isolates and affected patients of all age groups (Table 4). *C. albicans* infections occurred either alone (*n* = 69) or as mixed infection (*n* = 4) or as initial infection, followed by re-infection with *C. auris* (*n* = 1). *C. parapsilosis* (54 of 239, 22.6%), *C. tropicalis* (35 of 239, 14.6%), *C. auris* (33 of 239, 13.8%), and *C. glabrata* (32 of 239, 13.4%) were the second, third, fourth, and fifth most frequently isolated species, respectively. Other *Candida* species were isolated from 11 patients, including one patient co-infected with *C. krusei* and *C. glabrata*. The isolation frequency of *C. albicans* from neonates/infants was higher than *C. parapsilosis* and *C. glabrata,* and significantly higher (*p* < 0.05) than *C. tropicalis* and *C. auris*. *C. parapsilosis* (*n* = 54) was the most dominant non-*albicans Candida* species; similar to *C. albicans* and *C. tropicalis,* it was also isolated from all age groups. More importantly, *C. auris* was the fourth most common cause of candidemia in Kuwait in 2018, surpassing *C. glabrata,* and its isolation frequency among older (≥65 years old) patients was significantly higher than *C. albicans* (*p* = 0.001). 

The isolation frequency of *Candida* spp. from candidemia patients in all eight major and four tertiary care hospitals was also analyzed with respect to their point of care; the data are presented in Table 5. *C. albicans* was mostly (48 of 74, 64.9%) isolated from patients in the ICUs and was the dominant species (18 of 37, 48.6%) in Maternity Hospital as well as in Adan Hospital (15 of 38, 39.5%), with the latter also including maternity wards and neonatal ICUs catering mainly to the southern governorates within Kuwait. *C. parapsilosis* was the dominant species in only one major (Mubarak Al-Kabeer) hospital, while *C. tropicalis* was most frequently isolated from patients in two major (Amiri and Ibn-Sina) hospitals. More interestingly, in 2018, *C. auris* emerged as the dominant species in two major (Al-Sabah, 14 of 24, 58.3% and Farwaniya, 11 of 36, 27.8%) hospitals in Kuwait, surpassing even *C. albicans* and *C. parapsilosis*. Nine patients had fungemia due to other (*Cyberlindnera fabianii*, *n* = 4; *Magnusiomyces capitatus*, *n* = 2; *Kodamaea ohmeri*, *n* = 1; *Lodderomyces elongisporus*, *n* = 1; and *Rhodotorula minuta*, *n* = 1) yeast species.

### 3.2. AST Data and Molecular Basis of Antifungal Drug Resistance

The AST data against four (amphotericin B, fluconazole, voriconazole, and caspofungin) commonly used antifungal drugs for the five common *Candida* spp. are presented in Table 6. All *C. tropicalis* isolates were susceptible to all four antifungal drugs, while only one *C. albicans* isolate was resistant to fluconazole and voriconazole. Resistance to fluconazole among *C. parapsilosis* was high, as 9 of 54 (16.7%) isolates were resistant to this drug. As expected, many *C. parapsilosis* isolates also exhibited reduced susceptibility to caspofungin, but the MIC values were within the susceptible range. All *C. auris* isolates (*n* = 33) appeared susceptible to caspofungin (MIC range of 0.016–0.5 µg/mL) but were uniformly resistant to fluconazole, while six (18.1%) isolates were additionally resistant to voriconazole. Resistance to amphotericin B was also detected in 9 of 33 (27.3%) isolates. Resistance to fluconazole was also detected in 4 of 32 (12.5%) *C. glabrata* isolates. All four isolates also showed higher MIC (>0.5 µg/mL) to voriconazole and were considered resistant [14]. One *C. glabrata* isolate was intermediate to caspofungin and also showed reduced susceptibility (MIC of 0.095 µg/mL) to micafungin by Etest.

PCR sequencing data showed that the fluconazole-resistant *C. albicans* isolate (Kw150/8/18) contained two nonsynonymous mutations (T123I and Y132H) in addition to a few synonymous mutations in *ERG11*. Fluconazole resistance-conferring mutations (Y132F or K143R) in *ERG11* were detected in *C. auris* isolates. PCR sequencing studies of the HS-1 of the *FKS1* and *FKS2* genes showed that the *C. glabrata* isolate (Kw154/7/18), with reduced susceptibility to echinocandins, contained an S663P mutation in the HS-1 of *FKS2*. 

### 3.3. AST Data of Other Candida/Yeast Species, Clinical Details of Patients, Treatment, and Outcome

The AST data of 11 other *Candida* spp. and 9 other yeast species isolates are presented in Table 7. All four *C. krusei* isolates exhibited reduced susceptibility/resistance to fluconazole but were susceptible to voriconazole, amphotericin B, and caspofungin. Three *C. lusitaniae* and one *C. dubliniensis* isolates were uniformly susceptible to all four antifungal drugs (Table 7). Similarly, *C. blankii, C. guilliermondii,* and *C. pelliculosa* isolates were also susceptible to voriconazole, amphotericin B, and caspofungin but showed reduced susceptibility to fluconazole. Among nine other yeast species isolates, all *C. fabianii* (*n* = 4) and *L. elongisporus* were susceptible to all four antifungal drugs. Both *M. capitatus* isolates showed resistance to caspofungin, with one isolate also showing reduced susceptibility to fluconazole and amphotericin B. Both *K. ohmeri* and *R. minuta* isolates were resistant to fluconazole, with *R. minuta* showing additional resistance to caspofungin (Table 7). 

Fifteen of 20 patients with candidemia caused by other *Candida*/yeast species were admitted into the ICUs and their age varied from 5 days to 85 years (Table 7). The treatment details and outcome were available for 8 of 11 candidemia patients infected with other *Candida* species, and for 7 of 9 fungemia patients infected with other yeast species (Table 7). All three neonates infected with *C. lusitaniae* and one neonate with *C. pelliculosa* were successfully treated with liposomal amphotericin B alone, or with liposomal amphotericin B followed by caspofungin (Table 7). Similarly, one infant with *C. blankii* candidemia was also successfully treated with liposomal amphotericin B (Table 7). Overall, 2 of 8 (25%) patients infected with other *Candida* spp. died, including one patient co-infected with *C. krusei* and *C. glabrata* (Table 7). On the contrary, 5 of 7 (71.4%) fungemia patients due to rare yeast species died, including one patient who had received only a single dose of an antifungal drug and four patients in whom treatment was not even initiated as they succumbed to infection even before the culture produced a positive result (Table 7).

## 4. Discussion

Candidemia is the most common form of invasive candidiasis; however, it only represents nearly 75% of all invasive *Candida* infections. Furthermore, nearly 30% of all invasive *Candida* infections do not yield a positive blood culture [8,23,45]. Although the impact of candidemia and invasive candidiasis on morbidity, mortality, and healthcare costs is substantial, studies on candidemia on a population-based scale are challenging [6,8,9,10,14,46,47,48]. In this study, we determined the nationwide incidence of candidemia, the spectrum of *Candida* species, and their susceptibility to antifungal drugs in Kuwait in 2018. Kuwait is a small country located in the northwest portion of the Arabian Gulf in the Middle East. The country has a largely urban population that lives in the sprawling Kuwait City, with its suburbs divided into several governorates and two smaller towns nearly 45 Km away from Kuwait City; Ahmadi is located in the south and Jahra is located in the west. The total population of nearly 4.4 million individuals in 2018 comprised 1.34 million Kuwaiti nationals and 3.08 million expatriate workers or their dependents, mainly originating from south-southeast Asian, Middle Eastern, and African countries [49,50]. The expatriate work force in Kuwait in 2018 mostly comprised younger adults. Thus, the contribution of 30–59-year-old expatriate individuals was >68% of their total population, while the corresponding value for Kuwaiti nationals was ~31% (https://www.csb.gov.kw/Pages/Statistics_en?ID=67&ParentCatID=1, accessed on 14 July 2021). There are eight major and four tertiary care government hospitals located within a distance of ~45 Km from Kuwait City [51,52], which are where candidemia patients are diagnosed and treated in Kuwait. 

Our nationwide data on the incidence of candidemia in Kuwait in 2018 showed that 234 candidemic episodes occurred in 223 patients. The incidence of candidemia in Kuwait in 2018 was determined as 5.29 cases per 100,000 inhabitants, which is closer to the incidence in Israel and many European countries but lower than that reported from the United States of America (USA) [23,24,29,53,54]. A similar incidence rate has also been estimated for other nearby countries in the Middle East region [47]. Although most European countries have reported an incidence of candidemia between 1.4 and 5.7 cases per 100,000 inhabitants, Spain (8.1 cases per 100,000) and Denmark (>10 cases/100,000) have reported incidence values which are nearly 2–3 times that of the European average [8,23,24,46,55,56]. Similarly, studies from the USA have also reported higher incidence values, with a total of ≥7 cases per 100,000 inhabitants [23,24,54,57]. Epidemiological studies from China (26 cases per 100,000 hospital admissions compared to 90 cases per 100,000 patients in the USA) have also reported incidence values which are comparable to the data from Kuwait and European countries [23,24,58,59,60]. One study from Thailand, however, reported a higher incidence rate of 13.3 per 100,000 individuals [61]. The highest incidence rate of 89.1 cases per 100,000 was detected among neonates/infants, followed by 62.46 cases per 100,000 for the elderly (≥65 years old) in Kuwait, while the data from the USA showed the highest incidence rate of 20.1 cases per 100,000 among the elderly [54]. Only Spain has previously reported a higher incidence rate than Kuwait (96.4 cases per 100,000) for invasive candidiasis among children <1-year old [55], while Scotland has reported an incidence rate of 55.9 cases per 100,000 inhabitants among the elderly (≥65 years old), which is comparable to Kuwait [62]. Other European countries and the USA have reported incidence rates among children <1-year old or the elderly that were only 2–5 times higher than the national average [23,24,54]. This large difference between the total incidence rate and the incidence rate among neonates/infants or the elderly is likely due to the large number of 30-year-olds to 59-year-olds, mostly physically fit expatriate workers in Kuwait. This is consistent with the observations that the incidence of candidemia is dependent upon many factors such as the reference (total population, hospital admissions, or ICU admissions) used, the age of the patients (particularly the number of patients at the extremes of age), the overall health of the total population, and the number of patients with malignancies, transplants, or abdominal surgery [8,23].

Species distribution among candidemia patients showed that *C. albicans* was the predominant species but accounted for only 74 of 239 (31%) of all *Candida* spp. isolates, followed by *C. parapsilosis* (54 of 239, 22.6%) and *C. tropicalis* (35 of 239, 14.6%). The data showed that the contribution of *C. albicans* is steadily declining in Kuwait; from a high of 56% during the period of 1994–1998 to 41.8% between 2006 and 2011, and further to 33.1% during the period of 2012–2017 [18]. This decline continued in 2018 as well. A similar trend has also been noted in many other countries where non-*albicans Candida* species now account for the majority of invasive *Candida* infections [8,14,23,24,48,63]. On the contrary, one study from South Korea reported an increasing trend in the incidence of *C. albicans* candidemia in recent years [64]. The second most common *Candida* species isolated from patients with invasive candidiasis varies with geographical locations and is identified as *C. parapsilosis* in Latin/South America and many European and Middle Eastern countries, or as *C. glabrata* in North America, or as *C. tropicalis* in east/southeast Asian countries [8,14,23,24,65]. More recent studies have shown that *C. tropicalis* has now surpassed *C. albicans* as the predominant *Candida* species among candidemia patients in some south-southeast Asian countries [8,66,67]. An important finding of our study was the emergence of *C. auris* as the fourth most common *Candida* species isolated from candidemia patients, replacing *C. glabrata* and its emergence as the predominant species in two major (Al-Sabah and Farwaniya) hospitals in Kuwait. Furthermore, its isolation frequency among older (>65 years old) patients was higher than *C. glabrata* or *C. tropicalis* or *C. parapsilosis,* and significantly higher than *C. albicans*. The emergence of *C. auris* as a major bloodstream pathogen has dramatically changed the epidemiology of invasive candidiasis in many countries in recent years [22]. Unlike other *Candida* species, *C. auris* has caused major outbreaks in many healthcare centers around the world [22]. *C. auris* has now emerged as the predominant yeast pathogen in many healthcare centers and geographical locations [68,69,70,71].

Consistent with our previous studies [18,32], resistance to antifungal drugs among bloodstream *C. albicans* and *C. tropicalis* isolates was rare as only 1 of 74 *C. albicans* isolates exhibited in vitro resistance to fluconazole and voriconazole only. While the rate of resistance to fluconazole among *C. albicans* isolates in Kuwait is comparable to the worldwide data, fluconazole resistance among global *C. tropicalis* isolates is more common, particularly among isolates from east/southeast Asian countries [8,14,67]. The only triazole-resistant *C. albicans* isolate (Kw150/8/18) detected in this study contained two nonsynonymous (T123I and Y132H) mutations in *ERG11,* which are well-known to confer resistance of *C. albicans* to fluconazole [72]. Resistance of *C. parapsilosis* to fluconazole appears to be increasing consistently in Kuwait as 9 of 54 (16.7%) isolates in 2018 were resistant to this drug. Previously, only 1 of 310 (0.3%) and 21 of 446 (4.7%) *C. parapsilosis* isolates recovered in Kuwait during the periods from 2006 to 2011 and from 2012 to 2017, respectively, were detected as fluconazole-resistant strains [18]. Molecular genetic studies have shown that only ~40% of fluconazole-resistant *C. parapsilosis* isolates in Kuwait contain *ERG11* mutations, while the molecular basis of resistance in the remaining isolates remained unclear [39]. 

Consistent with previous reports [40,73], fluconazole resistance-conferring mutations (Y132F or K143R) in *ERG11* were also detected in the *C. auris* isolates described in this study. Although 31 *C. glabrata* isolates were susceptible, one isolate was intermediate to caspofungin. The latter isolate (Kw154/7/18) also exhibited reduced susceptibility to micafungin (MIC of 0.095 µg/mL) by Etest. PCR sequencing studies identified the S663P mutation in the HS-1 of *FKS2*. Clinical *C. glabrata* isolates are usually highly susceptible to micafungin (MIC values ≤ 0.03 µg/mL by both Etest and the broth microdilution-bases EUCAST method) [41,74]. This is the first report of the isolation of a bloodstream *C. glabrata* isolate in Kuwait showing reduced susceptibility to echinocandins and carrying the S663P mutation in the HS-1 of *FKS2*. Previously, the S663P mutation in the HS-1 of *FKS2* was only detected among non-blood (mainly urine) *C. glabrata* isolates from Kuwait [41]. Although the treatment history and outcome of infection were not available for this patient, our results support previous findings that *FKS* mutations are a better predictor of non-susceptibility of *C. glabrata* to echinocandins as compared to Etest [41,75].

Among 11 other *Candida* spp. isolates, *C. lusitaniae* isolates were susceptible to all four antifungal drugs tested, while isolates of *C. krusei, C. blankii, C. guilliermondii,* and *C. pelliculosa* exhibited reduced susceptibility to fluconazole but were susceptible to caspofungin, which is consistent with data reported in other studies [14,18,34,76,77]. Among other yeast species, *L. elongisporus* and *C. fabianii* isolates were susceptible to all four antifungal drugs, while *R. minuta* and *M. capitatus* isolates showed non-susceptibility to fluconazole and caspofungin. These data are also consistent with recent observations showing that rare *Candida*/yeast species exhibit non-susceptibility to antifungal drugs, including echinocandins [78,79,80]. 

The treatment details and outcome were available for 8 of 11 patients with other *Candida* and for 7 of 9 patients with rare yeast infections. Although the overall mortality among patients infected with other *Candida*/yeast species was 47%, higher mortality (71%) was seen among patients infected with rare yeast species. One of 2 patients with *C. krusei* infection died. This patient was also co-infected with *C. glabrata*. A higher mortality rate has been observed in candidemia patients infected with *C. krusei* as compared to those infected with *C. albicans*, *C. tropicalis,* or *C. parapsilosis* [81,82]. All three patients infected with *C. lusitaniae* were neonates who were successfully treated with liposomal amphotericin B, with/without additional treatment with caspofungin. The findings are similar to data reported for candidemia due to *C. lusitaniae* among neonates in Kuwait in a previous study [34]. Similarly, the neonate and infant infected with *C. pelliculosa* and *C. blankii* [42], respectively, were also successfully treated with liposomal amphotericin B. Amphotericin B has also been previously used successfully for the treatment of *C. pelliculosa* candidemia patients [83,84], while fluconazole treatment for *C. blankii* fungemia was not very effective (45% mortality) according to a recent study from India [85]. One of 3 fungemia patients infected with *C. fabianii* died even before culture results were obtained and treatment could begin, while all four patients infected with other rare yeasts expired, including three patients in whom blood cultures subsequently produced a positive result. A 20% mortality rate was also recently reported in an outbreak among neonates due to *C. fabianii* in Kuwait [86]. An overall mortality rate of 71% was seen among patients infected with rare yeast species. Our data are consistent with recent reports showing that fungemia due to rare yeast pathogens in both pediatric and adult patients is usually associated with mortality rates higher than those seen with common *Candida* species, particularly among patients receiving echinocandin therapy [82,87,88].

Our study has a few limitations. The candidemia incidence values described in this study were calculated based on the blood cultures received in the MRL. Since some blood cultures may not have been sent from individual hospitals to MRL, the actual candidemia incidence in Kuwait may be slightly higher. The antifungal susceptibility testing was performed by the Etest and not via the reference broth microdilution method. The clinical details, including the nationality, antifungal treatment given, and the outcome were not available for many patients.

In conclusion, the incidence of candidemia for the whole population of Kuwait was determined and was found to be 5.29 cases per 100,000 inhabitants. However, the incidence of candidemia showed wide variations among different population sections, with the highest incidence reported among neonates/infants, followed by the elderly (≥65 years old) subjects, while the lowest incidence was found among the 20–49-year-old group, largely due to the high number of younger and healthier expatriate workers in the country. Only 74 of 239 (31%) *Candida* spp. bloodstream isolates were identified as *C. albicans,* indicating that the vast majority (~70%) of candidemia cases in Kuwait are now caused by non-*albicans Candida*. Interestingly, *C. auris* has emerged as the fourth most common cause of candidemia, surpassing *C. glabrata,* and was also the predominant species in two major hospitals in Kuwait in 2018. Resistance to fluconazole appears to be increasing, particularly among *C. parapsilosis* isolates in Kuwait; *C. glabrata,* with reduced susceptibility to echinocandins, was also isolated for the first time from a candidemia patient in Kuwait. Infections with other *Candida*/other yeast species were also detected and were associated with high (>45%) mortality rates. This study provides an understanding of the epidemiology of invasive fungal infections in Kuwait and calls for the continuous monitoring of incidence and resistance trends as well as the emergence of clinically relevant yeast species, which may have a positive impact on patient care, infection control, and antifungal stewardship.

The DNA sequencing data reported in this study have been submitted to GenBank under accession no. LR137062 to LR137065, LS482924, MZ620708 to MZ620711, and MZ675666 to MZ675675. 

## Figures and Tables

**Table 1:** Number of beds, catchment area, and units/specialties available in different hospitals in Kuwait.

Hospital Name	Type of Facility	Catchment Area in Kuwait	No. of Beds	Major Units and/or Specialties Available
Adan	Secondary care	Ahmadi	826	MED, SUR, PAE, GAS, PUL, RHE, NEO, CAR, NEU, OBS, and GYN
KOC	Secondary care	Ahmadi	300	MED, SUR, PAE, GAS, PUL, RHE, and NEU
MAK	Secondary care	Hawally	726	MED, SUR, PAE, GAS, PUL, RHE, CAR, and NEU
Amiri	Secondary care	Capital	428	MED, SUR, GAS, PUL, RHE, CAR, and NEU
Al-Sabah ^a^	Secondary care	Central	372	MED, SUR, PUL, RHE, ENT, and OPT
NBK ^a^	Tertiary care	Entire Kuwait	67	P-HAE & P-ONC
Farwaniya	Secondary care	Farwaniya	868	MED, SUR, PAE, GAS, PUL, RHE, NEO, NEU, OBS, and GYN
Jahra	Secondary care	Jahra	765	MED, SUR, PAE, GAS, PUL, RHE, NEO, OBS, and GYN
Maternity	Specialized	Capital, Hawally	453	NEO, OBS, and GYN
Ibn Sina ^b^	Tertiary care	Entire Kuwait	355	Burn, NEU, NES, P-HAE, P-SUR, and KT
KCCC ^b^	Tertiary care	Entire Kuwait	199	A-HAE, A-ONC, and BMT
Chest	Tertiary care	Entire Kuwait	323	CAR, Cardiac, Pulmonary and Thoracic specialties
Al-Razi	Tertiary care	Entire Kuwait	465	Orthopedic

KOC, Kuwait Oil Company; MAK, Mubarak Al-Kabeer; NBK, National Bank of Kuwait; KCCC, Kuwait Cancer Control Center; MED, medical; SUR, surgical; PAE, pediatric; GAS, gastroenterology; PUL, pulmonology; RHE, rheumatology; NEO, neonatology; CAR, careology; NEU, neurology; OBS, obstetrics; GYN, gynecology; ENT, ear, nose, and throat; OPT, ophthalmology; P-HAE, pediatric hematology; P-ONC, pediatric oncology; NES, neurosurgery; P-SUR, pediatric surgery; KT, kidney transplantation; A-HAE, adult hematology; A-ONC, adult oncology; BMT, bone marrow transplantation. ^a^ Al-Sabah Hospital shares laboratory services with NBK Hospital. ^b^ Ibn Sina Hospital shares laboratory services with KCCC.

**Table 2:** The DNA sequences of forward and reverse primers and their location in *ERG11*.

Primer Name	Fragment Location	Nucleotide Position *	Direction	DNA Sequence
CalERG11F1	N-terminal fragment	−415 to −394	Forward	5′-CACGACAACTTTCAAAGATTGA-3′
CalERG11R1	N-terminal fragment	149 to 127	Reverse	5′-AATGGAGCTCTATCTTTTCTTAA-3′
CalERG11F2	Internal fragment 1	−93 to −71	Forward	5′-AAAGAAAGGGAATTCAATCGTTA-3′
CalERG11R2	Internal fragment 1	576 to 554	Reverse	5′-TTGAGTTTTCATAACATTGGCAA-3′
CalERG11F3	Internal fragment 2	440 to 462	Forward	5′-AATTTGCTTTGACTACTGATTCA-3′
CalERG11R3	Internal fragment 2	1043 to 1021	Reverse	5′-AAATCACCACCTTTTTCTTTCAA-3′
CalERG11F4	Internal fragment 3	909 to 931	Forward	5′-TATTCTTATGGGTGGTCAACATA-3′
CalERG11R4	Internal fragment 3	1445 to 1424	Reverse	5′-GTTCCCAATTGAACATAAGCAA-3′
CalERG11F5	C-terminal fragment	1315 to 1337	Forward	5′-TTTAACTCTTCTGATGAAGTTGA-3′
CalERG11R5	C-terminal fragment	1762 to 1739	Reverse	5′-ATTGAGTCATCCTAACAATTACAA-3′

* Nucleotide position is shown relative to the start codon (+1 nucleotide).

**Table 3:** Distribution of candidemia patients in eight major hospitals and other tertiary care hospitals in Kuwait in 2018.

Hospital	Total No. of	Gender		No. Candidemia of Patients of Different Age (years)					Hospital Unit	
	Patients	Male	Female	<1	≥1–19	≥20–49	≥50–64	≥65	ICU	Ward
Adan	38	24 ^a^	13 ^a^	10	3	10	2	13	22	16
Mubaral Al-Kabeer	36	17	19	0	5	3	4	24	5	31
Amiri	14	5	9	0	0	2	2	10	3	11
Al-Sabah	21	16	5	0	3	4	3	10	9 ^a^	11 ^a^
Ibn Sina	18	9	9	0 ^a^	2 ^a^	7 ^a^	4 ^a^	4 ^a^	10	8
Maternity	33	16	17	33	0	0	0	0	33	0
Jahra	15	8	7	2	7	1	1	4	8	7
Farwaniya	33	18	15	3	1	8	6	15	17	16
Others *	15	9	6	0	1	3	4	7	8	7

^a^ Details of one patient were not available. * Other hospitals included Ahmadi Kuwait Oil Company Hospital, Chest Diseases Hospital, Al-Razi Orthopedic Hospital, and Kuwait Cancer Control Center.

**Table 4:** Spectrum of *Candida* species isolated from candidemia patients in Kuwait in 2018.

Age (in years) of Candidemia Patients	*Candida* Species Isolates Identified as					
	*C. albicans, n* = 74	*C. parapsilosis,n* = 54	*C. tropicalis*, *n* = 35	*C. auris*, *n* = 33	*C. glabrata*, *n* = 32	Others ^a^, *n* = 11
<1	27	13	2	0	6	7
≥1–19	6	9	2	1	0	0
≥20–49	11	7	10	7	8	0
≥50–64	11	2	4	5	4	1
≥65	19	23	16	20	14	3
Unknown	0	0	1	0	0	0

^a^*C. krusei*, *n* = 4; *C. lusitaniae*, *n* = 3; *C. blankii*, *n* = 1; *C. dubliniensis*, *n* = 1; *C. guilliermondii*, *n* = 1; *C. pelliculosa*, *n* = 1.

**Table 5:** Distribution of *Candida* species among candidemia patients admitted into wards and ICUs of eight major and other hospitals in Kuwait.

Hospital Name	Unit	No. of Candidemic Episodes Caused by						Total
		*C. albicans*	*C. parapsilosis*	*C. tropicalis*	*C. auris*	*C. glabrata*	Other *Candida* Species ^a^	
Adan	ICU	12	2	1	1	6	0	22
	Ward	3	5	3	1	3	1	16
Mubarak Al-Kabeer	ICU	2	1	0	0	1	2	6
	Ward	11	13	7	0	4	1	36
Amiri	ICU	0	0	2	0	0	0	2
	Ward	3	1	5	2	3	0	14
Al-Sabah	ICU	1	1	1	8	1	0	12
	Ward	1	3	2	6	0	0	12
Ibn-Sina	ICU	2	2	3	3	0	0	10
	Ward	0	3	4	1	0	0	8
Maternity	ICU	18	8	0	0	4	4	34
	Ward	0	0	0	0	0	0	0
Jahra	ICU	2	3	2	0	1	1	9
	Ward	4	1	1	0	0	0	6
Farwaniya	ICU	5	2	2	7	1	1	18
	Ward	4	3	2	4	5	0	18
Others *	ICU	6	5	0	0	2	1	14
	Ward	0	1	0	0	1	0	2
Total		74	54	35	33	32	11	239

^a^*C. krusei*, *n* = 4; *C. lusitaniae*, *n* = 3; *C. blankii*, *n* = 1; *C. dubliniensis*, *n* = 1; *C. guilliermondii*, *n* = 1; *C. pelliculosa*, *n* = 1. * Other hospitals included: Ahmadi Kuwait Oil Company Hospital, Chest Diseases Hospital, Al-Razi Orthopedic Hospital, and Kuwait Cancer Control Center.

**Table 6:** Antifungal Susceptibility Data of *Candida* Species Isolates against Four Antifungal Drugs by Etest.

*Candida* Species	Antifungal Drug	MIC Range (μg/mL)	GM ± SD	Resistant, *n* (%)
*C. albicans* (*n* = 74)	Amphotericin B	0.012–0.19	0.05 ± 0.04	0
	Fluconazole	0.047–8	0.58 ± 1.14	1 (1.4)
	Caspofungin	0.003–0.19	0.06 ± 0.056	0
	Voriconazole	0.002–1	0.03 ± 0.13	1 (1.4)
*C. parapsilosis* (*n* = 54)	Amphotericin B	0.002–0.5	0.05 ± 0.13	0
	Fluconazole	0.19–256	1.43 ± 39.91	9 (16.7)
	Caspofungin	0.064–1.5	0.32 ± 0.2	0
	Voriconazole	0.002–1	0.04 ± 0.21	1 (1.9)
*C. tropicalis* (*n* = 35)	Amphotericin B	0.002–0.5	0.115 ± 0.123	0
	Fluconazole	0.19–1.5	0.7 ± 0.36	0
	Caspofungin	0.004–0.25	0.08 ± 0.06	0
	Voriconazole	0.012–0.19	0.07 ± 0.05	0
*C. auris* (*n* = 33)	Amphotericin B	0.047–2	0.855 ± 0.419	9 (27.3)
	Fluconazole	32–256	212.66 ± 62.13	33 (100)
	Caspofungin	0.016–0.5	0.24 ± 0.14	0
	Voriconazole	0.047–3	0.59 ± 0.89	6 (18.1)
*C. glabrata* (*n* = 32)	Amphotericin B	0.047–0.75	0.19 ± 0.2	0
	Fluconazole	3–256	13.91 ± 71.38	4 (12.5)
	Caspofungin	0.012–0.38	0.11 ± 0.07	1 * (3)
	Voriconazole	0.064–16	0.28 ± 2.92	4 (12.5)

* Intermediate; MIC, minimum inhibitory concentration; GM, geometric mean; SD, standard deviation.

**Table 7:** Demographic and clinical details of patients infected with other *Candida*/yeast species, susceptibility data, treatment given, and outcome.

Patient No.	Patients Details				Date of Onset of Fungemia	Isolate No.	*Candida* or Yeast spp.	Etest MIC (µg/mL) for				Antifungal Treatment	Outcome	Reference
	Hospital	Unit	Gender	Age				AMB	FLU	VOR	CFG			
1	MAK	Ward	Female	77 Years	18.01.2018	Kw217/1/18	*C. krusei*	0.25	8	0.125	0.25	CFG, 14 days	Discharged, 22.07.2018	This study
2	MAK	ICU	Female	58 Years	16.04.2018	Kw183/4/18	C. krusei **	0.75	48	0.25	0.25	CFG, 6 days	Expired, 24.04.2018	This study
3	Maternity	ICU	Female	5 Days	07.10.2018	Kw136/10/18	*C. krusei*	0.004	64	0.25	0.094	L-AMB, 14 days	Not available	This study
4	KOC	ICU	Male	73 Years	19.08.2018	Kw210/8/18	*C. krusei*	0.25	32	0.19	0.19	Not available	Not available	This study
5	Maternity	ICU	Male	15 Days	05.12.2018	Kw94/12/18	*C. lusitaniae*	0.016	0.25	0.012	0.032	L-AMB, 14 days	Discharged, 19.12.2018	This study
6	Farwaniya	ICU	Female	28 Days	30.12.2018	Kw39/1/2019	*C. lusitaniae*	0.016	0.38	0.016	0.008	L-AMB, 14 days; CFG, 15 days	Discharged, 18.02.2019	This study
7	Jahra	ICU	Male	9 Days	*n*. A.	Kw51/6/18	*C. lusitaniae*	0.047	0.25	0.006	0.125	L-AMB, 21 days	Discharged, 29.07.2018	This study
8	Maternity	ICU	Male	4 Months	14.03.2018	Kw142/3/18	*C. blankii*	0.125	12	0.38	0.25	L-AMB, 14 days	Discharged, 06.12.2018	[42]
9	Adan	Ward	Male	5 Months	17.05.2018	Kw205/5/18	*C. guilliermondii*	0.032	4	0.094	0.38	Not available	Not available	This study
10	Maternity	ICU	Male	3 Days	21.05.2018	Kw251/5/2018	*C. pelliculosa*	0.023	6	0.19	0.032	L-AMB, 14 days	Discharged, 06.12.2018	This study
11	MAK	ICU	Female	76 Years	14.08.2018	Kw152/8/18	*C. dubliniensis*	0.016	0.38	0.064	0.01	CFG, 3 days	Expired, 26.08.2018	This study
12	Maternity	ICU	Male	1 Month	14.03.2018	Kw80/4/18	*C. fabianii*	0.25	2	0.125	0.38	L-AMB + CFG, 14 days; FLU + CFG, 14 days	Discharged, 28.05.2018	This study
13	Amiri	ICU	Male	54 Years	26.06.2018	Kw146/7/18	*C. fabianii*	0.5	2	0.064	0.094	None *	Expired, 27.06.2018	This study
14	Maternity	ICU	Male	8 Days	28.07.2018	Kw303/7/18	*C. fabianii*	0.064	6	0.19	0.047	L-AMB, 14 days	Not available	This study
15	Maternity	ICU	Male	3 Months	09.09.2018	Kw106/9/18	*C. fabianii*	0.5	1.5	0.094	0.094	L-AMB, 6 weeks	Discharged, 27.03.2019	This study
16	MAK	ICU	Female	67 Years	20.09.2018	Kw159/9/18	*M. capitatus*	1.5	12	0.5	32	None *	Expired, 21.09.2018	[43]
17	Amiri	ICU	Female	85 Years	03.06.2018	Kw86/6/18	*M. capitatus*	0.5	3	0.19	32	None *	Expired, 06.06.2018	[43]
18	MAK	Ward	Female	71 Years	21.02.2018	Kw261/2/18	*L. elongisporus*	0.012	0.125	0.004	0.064	CFG, One dose only	Expired, 22.02.2018	[44]
19	Amiri	Ward	Female	79 Years	25.10.2018	Kw25/11/18	*K. ohmeri*	0.008	256	0.19	0.19	None *	Expired, 27.10.2018	This study
20	Adan	Ward	Female	28 Days	29.10.2018	Kw162/11/18	*R. minuta*	6	256	2	32	Not available	Not available	This study

AMB, amphotericin B; FLU, fluconazole; VOR, voriconazole; CFG, caspofungin; L-AMB, ambisome; *n*.A., not available. * No treatment was given as the patients expired before culture produced a positive result. ** Mixed infection with *C. glabrata.*

## Data Availability

All the data are available in the published article; the datasets are available from the corresponding author and can be provided upon reasonable request.
